# Homelessness and mortality: gender, age, and housing status inequity in Korea

**DOI:** 10.4178/epih.e2024076

**Published:** 2024-09-12

**Authors:** Gum-Ryeong Park, Dawoon Jeong, Seung Won Lee, Hojoon Sohn, Young Ae Kang, Hongjo Choi

**Affiliations:** 1 Dalla Lana School of Public Health, University of Toronto, Toronto, ON, Canada; 2 Department of Health, Aging, & Society, McMaster University, Hamilton, ON, Canada; 3 Department of Preventive Medicine, Seoul National University College of Medicine, Seoul, Korea; 4 Institute for Immunology and Immunological Disease, Yonsei University College of Medicine, Seoul, Korea; 5 Department of Human Systems Medicine, Seoul National University College of Medicine, Seoul, Korea; 6 Institute of Health Policy and Management, Seoul National University Medical Research Center, Seoul, Korea; 7 Division of Pulmonary and Critical Care Medicine, Department of Internal Medicine, Severance Hospital, Yonsei University College of Medicine, Seoul, Korea; 8 Division of Health Policy and Management, Korea University, Seoul, Korea

**Keywords:** Homelessness, Housing, Mortality, Age, Gender, Korea

## Abstract

**OBJECTIVES:**

We compared mortality rates among various housing statuses within the homeless population and investigated factors contributing to their deaths, including housing status, gender, and age.

**METHODS:**

Using a comprehensive multi-year dataset (n=15,445) curated by the National Tuberculosis Screening and Case Management Programs, matched with the 2019-2021 Vital Statistics Death Database and National Health Insurance claims data, we calculated age-standardized mortality rates and conducted survival analysis to estimate differences in mortality rates based on housing status.

**RESULTS:**

The mortality rate among the homeless population was twice as high as that of the general population, at 1,159.6 per 100,000 compared to 645.8 per 100,000, respectively. Cancer and cardiovascular diseases were the primary causes of death. Furthermore, individuals residing in shelter facilities faced a significantly higher risk of death than those who were rough sleeping, with an adjusted hazard ratio of 1.70 (95% confidence interval, 1.37 to 2.11). This increased risk was especially pronounced in older adults and women.

**CONCLUSIONS:**

The study highlights the urgent need for targeted interventions, as the homeless population faces significantly higher mortality rates. Older adults and women in shelter facilities are at the highest risk.

## GRAPHICAL ABSTRACT

[Fig f3-epih-46-e2024076]

## Key Message

The mortality rate of the general population has significantly declined, leading to an increase in mortality inequality between the homeless and the general population from 1.3 to 1.8 times. In particular, the mortality risk for homeless individuals in facilities was higher than that of those living in *jjokbang* or on the streets. This study highlights the need for a policy shift to promote deinstitutionalization for the homeless population.

## INTRODUCTION

The homeless population is characterized by a lack of stable and adequate housing, which exposes them to numerous health risks and socioeconomic barriers [1]. The absence of suitable housing and employment opportunities not only undermines their living standards [2], but also increases the likelihood of these individuals experiencing distressing life events, including discrimination and victimization related to criminal activities. Additionally, the lack of access to structured social and health care services impedes their ability to remain resilient against both external and internal stressors [3,4]. This combination of multifaceted barriers can, in turn, expose the homeless to increased risks of diseases and adverse outcomes from illnesses, including mortality [5]. Much of the existing literature provides evidence that the homeless, compared to the general population, experience higher rates of infectious diseases (e.g., tuberculosis [TB]) [6], psychiatric morbidities [7], and premature mortality [2,5], even in settings with universal healthcare and insurance systems [8].

While the term “homeless” was initially defined by the lack of stable, adequate housing, this group is diverse in demographics and characteristics, which further illustrates the heterogeneity of this often overlooked population [8]. UN-Habitat recognizes that homelessness can take various forms, ranging from individuals living on the streets or in open spaces to those residing in temporary accommodations and informal settlements. First, within the homeless population, individuals who lack conventional housing or live in temporary shelters are at a higher risk of mortality. This increased risk is partly due to (1) widespread cumulative exposures and susceptibility to illness in unregulated and unsanitary conditions, and (2) the fact that temporary shelters often provide limited, tailored healthcare and social services [9,10]. Second, some homeless individuals may reside in temporary housing that includes support services such as counseling and healthcare, typically offered by non-profit organizations or publicly funded agencies. In these settings, case managers play a crucial role in assisting residents to adhere to plans aimed at improving their living standards, often by enforcing rules and regulations [11].

Prior studies have indicated that the relationship between homelessness and mortality varies according to demographic factors such as gender and age. Some researchers have argued that women may be more susceptible to threatening environments, including the risk of sexual assault, which could lead to a greater vulnerability to diseases and illnesses [2,5,12]. Others have suggested that engaging in unhealthy behaviors, such as substance abuse and alcohol consumption, may contribute to heightened mortality risks among homeless men. Second, homeless individuals are not immune to age-related risk factors [5,13], encountering conditions such as frailty and cognitive impairment that are typically associated with older age groups [2]. Drawing on the concept of intersectionality, which emphasizes the interconnected nature of social identities [14], this evidence indicates that homelessness, age, and gender can interact in complex ways to affect the health of homeless individuals. For instance, older homeless men may experience higher mortality rates compared to older homeless women, due to a combination of age-related health vulnerabilities and gender-specific risks such as higher rates of unhealthy behaviors, which can worsen health conditions and increase the risk of mortality [15].

Korea has experienced a significant increase in its homeless population, a trend significantly worsened by the 1998 Asian economic crisis, which led to widespread mass layoffs [16]. Despite the implementation of the Homeless Welfare Act, which requires the provision of health and social care support for those experiencing homelessness, their living conditions still pose a considerable risk to their overall health and well-being [16,17]. In terms of housing, shelter facilities are the primary residential option available to homeless individuals. Although these facilities are essential for providing immediate relief, such as temporary shelter and support services, they fall short of fully meeting the basic needs and delivering customized healthcare and non-healthcare services to the homeless [11].

In the Korean context, another layer of sheltered homelessness, known as “*jjokbang*,” is characterized by informal settlements [18]. Although *jjokbang* shares similarities with shelters and facilities in terms of substandard sanitation and shared amenities such as toilets, it is somewhat unique in that it offers residents a degree of autonomy, allowing them to plan their daily activities voluntarily [19]. Originally established as dormitories for nearby factories, *jjokbang* often served as provisional accommodations for individuals displaced during the Korean War. These facilities later underwent renovations to accommodate those without a permanent residence. Despite these changes, they remain unregulated, with substandard physical conditions, inadequate sanitation facilities, and provide single-room occupancy with a size of 3 square meters [20]. This is due to governmental measures aimed at marginalizing these housing forms within the context of urban beautification and redevelopment initiatives [21]. A significant number of individuals grappling with homelessness turn to *jjokbang* as the last residential safety net before officially entering a state of homelessness or as a transitional means to exit from it [18]. While *jjokbang* currently serves as a temporary refuge for individuals facing the hardships of rough sleeping, it falls short of providing sufficient accommodations [20].

Several studies have explored the lives and health conditions of homeless individuals in Korea. Homeless populations in this country are exposed to various risks, including infectious diseases (e.g., TB) [6], mental health problems [22], and issues related to alcohol consumption [23,24]. Additionally, residents of *jjokbang* suffer from extreme weather conditions due to inadequate heating and cooling systems [18], which increases their risk of preventable mortality. This evidence underscores the urgent need for comprehensive strategies to address the housing and health needs of homeless individuals. However, the existing literature primarily focuses on specific regions and captures data at single time points, raising concerns about the representativeness and generalizability of these findings. More critically, because homeless individuals often face issues of invisibility and mobility, longitudinal tracking provides essential insights into the factors that affect their survival. Furthermore, to the best of our knowledge, there are currently no studies that offer a comprehensive national overview of the homeless population or investigate the mortality risks associated with their living conditions.

To address these knowledge gaps, we aim to leverage a comprehensive database that encompasses data from a large-scale, multiyear nationwide TB active case finding program specifically targeting the homeless population. Our goal is to investigate the differences in mortality risks within the homeless population and in comparison to the general population in Korea. Specifically, we examined the intricate issue of homelessness in Korea, considering how baseline housing conditions, along with gender and age, influence mortality risks among the homeless.

## MATERIALS AND METHODS

### Data and study participants

This research utilizes data from three distinct sources: the nationwide TB screening and case management programs targeting vulnerable populations, including homeless people and migrants in Korea, and the 2019-2021 Vital Statistics Death Database (VSDD). Initiated as a pilot project in 2019, the Korea Disease Control and Prevention Agency (KDCA), in collaboration with the Korean National Tuberculosis Association (KNTA), has implemented comprehensive TB screening and case management initiatives specifically for the homeless population in Korea [25]. These programs, managed by regional KNTA branches, involved history-taking questionnaires and remote interpretations of chest X-rays by radiologists. Patients with significant TB findings on chest X-rays were categorized as either suggested TB cases requiring further evaluation or as having inactive TB, while others were classified as normal or diagnosed with other lung diseases. On-site sputum collection for acid-fast bacillus smears, molecular tests, and cultures was conducted for individuals showing presumptive signs of TB. Vehicles equipped with chest X-ray facilities screened street homeless individuals and residents of homeless facilities, while ultraportable devices were used for *jjokbang* residents.

**Figure 1.**
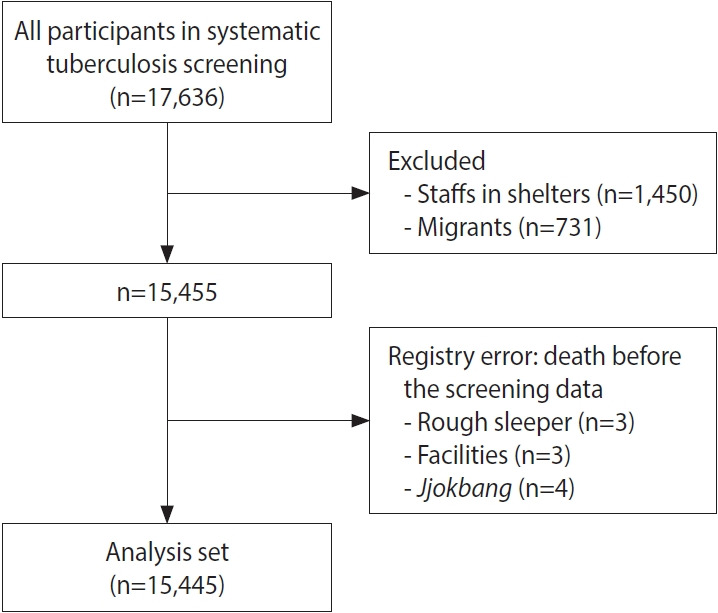
Flow diagram of the study participants.

The dataset derived from these programs encompasses a broad spectrum of demographic data, including age and gender, as well as TB-specific information such as the individual’s TB history and associated symptoms. Additionally, it includes radiological diagnostic findings related to TB, the frequency of screening procedures, and health-related information, such as smoking habits. The VSDD acts as an administrative repository for the primary cause of death and the timing of deaths for all registered deaths in Korea. By utilizing individual-level anonymous identifiers, a crucial linkage has been established between individual TB screenings, health insurance claim records, and death records. These identifiers, assigned to all residents in Korea, facilitate the precise tracking and integration of health screening data with mortality information. Moreover, data from the national health insurance claim database are utilized to generate variables for comorbidities. Out of the 17,636 participants in the case-finding programs, we successfully linked 15,445 individuals with health-related information, excluding ineligible samples (e.g., 1,450 staff in shelters and 731 migrants) and observations with registry or coding errors (10 individuals) (Figure 1). Ultimately, we constructed a retrospective homeless cohort. This linkage enables the creation of comprehensive individual-level combined datasets, which allow for the tracking and analysis of mortality trends over time.

#### Independent variable

The housing status of homeless individuals was determined by their current living conditions, or the absence thereof, characterized by a lack of stable, permanent residence. This status was categorized into 3 groups: rough sleepers, sheltered homeless individuals (facility residents), and jjokbang residents.

#### Dependent variable

The dependent variable in this study was the vital/mortality status of the participants during our follow-up period, categorized as either deceased or survived. We defined survival duration as the number of years from the baseline assessment to the occurrence of death, as recorded in the VSDD, or until the end of the followup period on October 19, 2022. Given potential delays in the reporting of cause-of-death statistics, we included death notifications up to December 31, 2022, to accurately determine the causes of death. In the TB Active Case Finding (ACF) Program, participants may undergo screening multiple times over different years or at specific intervals, such as biannually. We based the baseline characteristics on data collected at the initial screening date.

#### Covariates

In this study, we considered a comprehensive range of control variables. Demographic characteristics included gender and age, with age divided into four categories: 54 or younger, 55 to 64, 65 to 75, and over 75. TB-related information encompassed the year of the first screening (categorized), TB history, X-ray results (either normal or abnormal), the number of TB screenings (ranging from 1 to 4) during the study period, and TB diagnosis status (either yes or no) at each screening. For health and health behavior variables, we included TB-related symptoms such as coughing, along with participants’ smoking and alcohol consumption histories. Following guidelines [26], we employed the Charlson comorbidity index, which ranges from 0 to 3 or above. This index, derived from a combination of medical records and claim data, was used to account for variations in participants’ comorbidities, excluding those related to TB.

### Statistical analysis

First, we estimated the crude mortality rate and standardized mortality rate (SMR) for both the study’s homeless population and the general population to assess the differences. The SMR was calculated as the ratio of the observed number of deaths to the expected number of deaths, adjusted for the age distribution of the 2005 general population in Korea. We selected this reference group to ensure standardization and comparability of mortality calculations across different time periods. To determine if the mortality rates of the homeless population significantly differed from those in the general population, we compared our estimates with those of the Seoul metropolitan homeless population in 2005 [27] and the general population in Korea for the years 2005 and 2020, as reported in the Organization for Economic Cooperation and Development database. We also provided a breakdown of the causes of death based on the available information for the homeless individuals who died during the follow-up period. Next, we utilized a Cox proportional hazard model to estimate whether housing status is associated with differences in mortality rates, after controlling for a range of explanatory variables. We conducted a gender-stratified analysis to estimate the mortality risk associated with housing status for men and women separately. Subsequently, we performed an age-stratified analysis to explore the potential impact of age on the relationship between housing status and mortality.

### Ethics statement

This study was approved by the Institutional Review Board of Severance Hospital (4-2022-0595).

## RESULTS

Table 1 presents the mortality rates by gender and housing status among the homeless population. The SMR among homeless individuals was 1,159.6, which was approximately twice as high as that of the general population in Korea (635.8 deaths per 100,000 population in 2020). When analyzing mortality rates by gender, disparities persisted between homeless individuals and the general population. Homeless men experienced a significantly higher excess number of deaths (1,346.9-849.9= 497 deaths per 100,000) than homeless women (519.1-483.5= 35.6 deaths per 100,000). In terms of housing status, rough sleepers exhibited the highest SMR (1,242.1 deaths per 100,000), followed by those residing in *jjokbang* (1,176.5 deaths per 100,000) and those in shelter facilities (1,098.4 deaths per 100,000). Across all housing statuses, men had a higher mortality rate than women. Descriptive statistics of the study population are provided in Supplementary Material 1.

**Figure 2.**
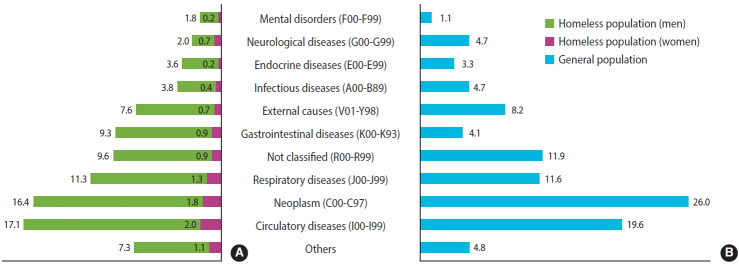
Cause of deaths between homeless population (A) and the general population (B). The cause of death for the general population from cause-of-death statistics in 2021 in Korea.

Figure 2 illustrates that the causes of death among homeless individuals were largely similar to those in the general population. The primary causes of death among the homeless were circulatory diseases (19.1%) and neoplasms (18.2%), which closely align with the leading causes in the general population, where neoplasms are the most prevalent, followed by circulatory diseases. However, a significant disparity was noted in the incidence of gastrointestinal diseases, accounting for 10.2% of deaths among the homeless compared to only 4.1% in the general population. No significant differences were observed in the trends of cause-of-death proportions between genders.

Supplementary Material 2 presents distinct patterns in the leading causes of death among rough sleepers, varying by gender. In men, circulatory diseases (27.6%) and causes not classified (17.1%) were the most prevalent, whereas external causes accounted for one-third of the deaths among women. In contrast, among individuals residing in shelter facilities, the primary causes of death showed different trends, with neoplasms (22.4%) and respiratory diseases (18.4%) being the most common. Within the specific subgroup of individuals living in *jjokbang*, the major causes of mortality also varied by gender. For men, neoplasms (19.2%), circulatory diseases (18.6%), and gastrointestinal diseases (16.0%) were the leading causes, while women experienced a higher prevalence of circulatory diseases (28.6%) and causes not classified (21.4%). Notably, one-fourth of deaths from infectious diseases were attributed to TB (1.1%), and more than half of the deaths from gastrointestinal diseases were linked to liver disease (5.8%) (Supplementary Material 3).

As shown in Table 2, we estimated the association between homelessness and mortality. Model 1 shows that facility residents had a higher mortality risk than rough sleepers (hazard ratio [HR], 1.70; 95% confidence interval [CI], 1.37 to 2.11). However, there was no statistically significant difference in mortality risk between rough sleepers and *jjokbang* residents. In model 2, which focused on men, both shelter and *jjokbang* residents demonstrated an increased mortality risk, with HRs of 1.56 (95% CI, 1.24 to 1.95) and 1.18 (95% CI, 0.95 to 1.46), respectively. Nevertheless, the mortality risk for *jjokbang* residents was similar to that of rough sleepers. A similar pattern was observed for women in model 3, where the HR for shelter residents was 5.01 (95% CI, 1.92 to 13.06). Again, no significant difference in mortality risk was found between rough sleepers and *jjokbang* residents.

Table 3 presents the results of an age-stratified analysis among homeless men. In men under the age of 65, no significant association was found between the category of homelessness and mortality (model 1). However, model 2 revealed a significant increase in mortality risk for facility residents compared to rough sleepers among the older adult population, specifically those aged 65 and older (HR, 2.20; 95% CI, 1.52 to 3.18). No significant differences in mortality risks were observed between rough sleepers and *jjokbang* residents.

## DISCUSSION

The present study aimed to investigate mortality risks among the homeless population in Korea. Specifically, we seek to identify disparities in mortality based on housing status, gender, age, and primary causes of death within this population.

First, the observed mortality rate is significantly lower than the rate recorded in 2005, which was 1,311 deaths per 100,000, even though the earlier estimates were confined to the Seoul metropolitan area, where most of our study population still resides. These findings suggest that a series of policies aimed at addressing homelessness have been effective in reducing mortality risks. For example, in response to a significant increase in the homeless population, both government and non-government organizations have actively implemented a variety of strategies. These include financial, healthcare, and social support services, as well as the establishment of emergency shelters and the initiation of community outreach programs, following the enactment of the “Act on Support for Welfare and Self-Reliance of the Homeless” on June 7, 2011. Supporting this, previous research has shown that such programs are effective in promoting independent living and improving the health of Korean homeless individuals [28,29]. A particularly striking example of this success is the marked reduction in mortality from infectious diseases such as tuberculosis, with rates falling from 9.6% in 2005 to 4.2% in our study. This decline is likely due to the comprehensive national TB programs [6,27].

Despite significant progress in addressing homelessness in Korea, it is important to recognize that homeless individuals still face higher mortality risks. Compared to the general population, there was an excess of 523 deaths per 100,000 among the homeless. This finding is consistent with previous research [2,5], which highlights substantial health disparities between homeless individuals and the general population. These disparities can be attributed to several factors, including unequal access to healthcare services and precarious living conditions that expose them to increased risk factors. Such exposures may contribute to heightened mortality risks [1,5]. The primary causes of death among the homeless are not only cancer and cardiovascular disease but also external causes, which could be addressed with timely and appropriate healthcare. Building on existing research on healthcare approaches for homeless people [30], it is clear that prioritizing access to healthcare systems could be a crucial strategy to reduce the mortality risks faced by this population.

Another finding is that age and gender significantly influenced the mortality risks faced by homeless individuals. The link between housing status and mortality risks is particularly strong among older homeless individuals, who have unique health and social care needs compared to their younger counterparts, largely due to the aging process. Additionally, their reduced functionality and mobility can hinder access to necessary services [31]. Regarding gender, although homeless women have lower mortality rates than homeless men, the disparities in mortality by housing status are more pronounced among women. This indicates that a wider array of risk factors may more actively affect the mortality risks for homeless women, potentially making them more susceptible to various health challenges linked to housing status.

Lastly, the adjusted risks of death were higher among sheltered homeless individuals, while the risks for rough sleepers and those living in *jjokbang* were relatively similar. This finding contradicts previous research, which suggested that rough sleepers—those without conventional accommodations—face the highest mortality risks among different homeless groups [9]. The discrepancies might be due to selection bias, as rough sleepers who died early may not have been included in the study, or due to reverse causation, where sicker individuals are more likely to stay in shelters [32]. However, since sheltered homeless individuals are typically younger and have fewer comorbidities than residents of *jjokbang*, the likelihood of reverse causation is reduced. Therefore, issues related to institutionalization and the limited autonomy of vulnerable populations could lead to diminished health rights and poorer health outcomes. For those seeking to maintain an independent lifestyle, *jjokbang* may represent their ultimate housing choice. Nevertheless, it is crucial to acknowledge that *jjokbang* does not equate to decent housing, as it often features substandard living conditions, including poor ventilation, inadequate sanitation, and insufficient heating [20].

These findings have significant policy implications. First, it is crucial to recognize the importance of adopting long-term, recovery-oriented approaches. In Korea, local governments provide housing support programs that primarily offer temporary accommodations. These programs often require individuals to save a minimal deposit to qualify, which may exclude those without a minimum income. Additionally, the limited availability of these programs restricts access and participation opportunities. Valuable insights can be drawn from initiatives like North America’s Housing First approach, which prioritizes harm reduction by offering homeless individuals permanent housing and psychological support without initial conditions [33,34]. Therefore, relevant organizations should (1) identify the challenges and barriers to recovery and (2) prioritize the provision of permanent housing to support the independent living of homeless individuals. Second, considering the significant mortality gaps observed among older adults and women in our study, interventions should be tailored to meet their specific health, social, and housing needs. This includes providing specialized geriatric healthcare services, gender-specific healthcare, and ensuring secure housing environments. These initiatives align with the principles of deinstitutionalization, which advocate for a transition from large, centralized institutions or shelters to community-based care and supportive housing solutions. This shift promotes replacing temporary and restrictive housing arrangements with long-term options that enhance the independence and well-being of socioeconomically disadvantaged individuals.

There are some limitations in this study. First, the baseline data collection was conducted at a single screening point for TB among individuals experiencing homelessness. Therefore, our findings may not provide a comprehensive assessment of the impact of time-varying conditions, such as changes in housing status, on mortality within this population. Additionally, it is important to consider that individuals may transition in and out of homelessness during the follow-up period, which could potentially lead to an underestimation of mortality risks. Second, although our analysis focused on examining mortality disparities among different types of homelessness, we must acknowledge the potential heterogeneity within the baseline population. For instance, our sampling method may have selectively included healthier rough sleepers, while excluding less healthy ones who are less visible within the homeless community. Future studies may wish to explore the nature of services or programs available to assist homeless individuals in reducing their mortality risks. Despite these limitations, it is important to recognize the strengths of this study. Notably, this is one of the first investigations to monitor mortality over an extended period. Moreover, this study utilizes a substantial dataset obtained from large-scale TB screenings of homeless individuals. Given that much of the prior research has largely relied on data from specific, localized homeless populations, our study’s findings make a valuable contribution in terms of their potential generalizability to the broader homeless population in Korea.

## Figures and Tables

**Figure f3-epih-46-e2024076:**
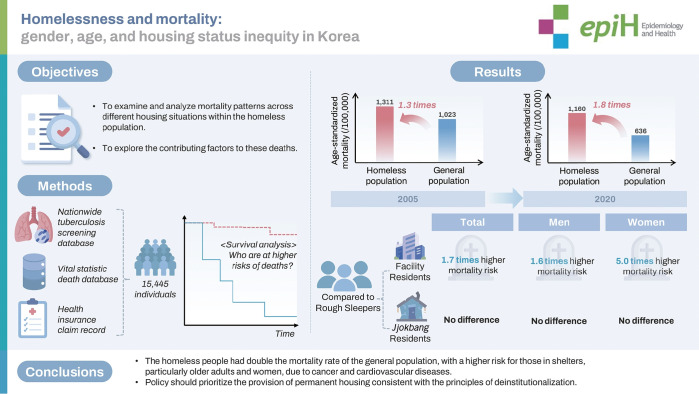


**Table 1.** Person-years and mortality rate by homelessness type and gender

Variables	n	Person-year	Mortality rate (per 100,000)					
			Crude	Standardized (95% CI)	General population (2005)^1^		General population (2020)^1^	
					Crude	Standardized	Crude	Standardized
Total	15,445	30,064	2,787.4	1,159.6 (1,033.1, 1,286.0)	510.3	1,023.1	588.3	635.8
Men	12,256	23,660	3,186.8	1,346.9 (1,200.6, 1,493.1)	558.2	1,328.1	636.6	849.9
Women	3,089	6,404	1,311.7	519.1 (265.0, 773.1)	461.8	806.9	541.1	483.5
Rough sleeper	3,192	5,746	2,645.3	1,242.1 (961.4, 1,522.7)	-	-	-	-
Men	2,896	5,236	2,769.3	1,345.9 (1,039.1, 1,652.7)	-	-	-	-
Women	296	510	1,372.5	353.3 (0.0, 766.4)	-	-	-	-
Facilities	7,081	14,821	2,523.4	1,098.4 (931.8, 1,265.0)	-	-	-	-
Men	5,161	10,699	3,028.3	1,319.5 (1,117.5, 1,521.4)	-	-	-	-
Women	1,920	4,122	1,213.0	621.8 (302.7, 941.0)	-	-	-	-
*Jjokbang*	5,172	9,505	3,282.5	1,176.5 (981.8, 1,371.2)	-	-	-	-
Men	4,199	7,731	3,686.5	1,365.1 (1,141.9, 1,588.4)	-	-	-	-
Women	973	1,774	1,522.0	196.2 (110.4, 281.9)	-	-	-	-

CI, confidence interval.

1 From the Organization for Economic Cooperation and Development database.

**Table 2.** Housing status and mortality according to gender

Variables	Model 1	Model 2	Model 3
Gender	Total	Men	Women
Age	Total	Total	Total
Rough sleeper	1.00 (reference)	1.00 (reference)	1.00 (reference)
Facilities	1.70 (1.37, 2.11)^***^	1.56 (1.24, 1.95)^***^	5.01 (1.92, 13.06)^*^
*Jjokbang*	1.10 (0.89, 1.36)	1.18 (0.95, 1.46)	1.04 (0.43, 2.50)
Total (n)	15,445	12,256	3,189

Values are presented as hazard ratio (95% confidence interval). All models controlled for gender, age, the first screening year, tuberculosis (TB) history, cough history, chest radiography results, smoking history, number of screenings, TB diagnosis, and Charlson comorbidity index.

* p<0.05,

*** p<0.001.

**Table 3.** Housing status and mortality according to age among men

Variables	Model 1	Model 2
Age (yr)	<65	≥65
Men		
Rough sleeper	1.00 (reference)	1.00 (reference)
Facilities	1.16 (0.86, 1.55)	2.20 (1.52, 3.18)^***^
*Jjokbang*	1.15 (0.87, 1.52)	1.13 (0.93, 1.88)
Total (n)	8,828	3,428

Values are presented as hazard ratio (95% confidence interval). All models control for the first screening year, tuberculosis (TB) history, cough history, chest radio graphy results, smoking history, number of screenings, TB diagnosis, and Charlson comorbidity index.

*** p<0.001.

## Supplementary materials

Supplementary Material 1. Demographic and socioeconomic characteristics by gender and housing status

Supplementary Material 2. Share of cause of death by gender and housing status

Supplementary Material 3. Specific causes of death by gender and housing status
