# Supplementary material for: Homelessness and mortality: gender, age, and housing status inequity in Korea
Source: Epidemiol Health. 2024 Sep 12;46:e2024076. doi: 10.4178/epih.e2024076 (PMC11826014; doi:10.4178/epih.e2024076)
Supplement: Supplementary Material 1. — Demographic and socioeconomic characteristics by gender and housing status [file epih-46-e2024076-Supplementary-1.docx]

## SupplementaryMaterial 1. Demographic and socioeconomic characteristics by gender and housing status

|  | Men | | | Women | | |
| --- | --- | --- | --- | --- | --- | --- |
|  | Rough sleeper | Facilities | Jjokbang | Rough sleeper | Facilities | Jjokbang |
| Age |  |  |  |  |  |  |
| 0-54 | 43.16% | 36.41% | 27.08% | 22.30% | 33.54% | 12.13% |
| 55-64 | 34.60% | 37.94% | 38.15% | 22.30% | 36.77% | 19.01% |
| 65-74 | 14.12% | 20.17% | 25.22% | 25.00% | 20.94% | 33.40% |
| 75+ | 8.11% | 5.48% | 9.55% | 30.41% | 8.75% | 35.46% |
| Region |  |  |  |  |  |  |
| Seoul | 81.15% | 34.72% | 54.08% | 51.69% | 17.08% | 45.32% |
| Daegu | 3.80% | 7.98% | 11.96% | 1.69% | 13.91% | 12.74% |
| Busan | 3.11% | 2.81% | 20.65% | 16.22% | 3.13% | 17.57% |
| Daejeon | 5.04% | 3.16% | 10.22% | 25.34% | 1.77% | 14.08% |
| Incheon | 0.28% | 5.21% | 3.10% | 0.34% | 4.90% | 10.28% |
| Others | 6.63% | 46.12% | 0.00% | 4.73% | 59.22% | 0.00% |
| TB history |  |  |  |  |  |  |
| No history | 86.46% | 78.51% | 81.64% | 93.24% | 82.34% | 89.72% |
| Unknown | 2.49% | 8.22% | 6.67% | 3.72% | 9.90% | 5.65% |
| Previously treated | 11.05% | 13.27% | 11.69% | 3.04% | 7.76% | 4.62% |
| Chest radiography |  |  |  |  |  |  |
| Normal | 80.08% | 83.57% | 81.21% | 93.24% | 91.93% | 90.65% |
| Abnormal | 18.06% | 15.54% | 16.12% | 5.74% | 7.45% | 8.74% |
| Missing | 1.86% | 0.89% | 2.67% | 1.01% | 0.63% | 0.62% |
| Smoking |  |  |  |  |  |  |
| Never | 31.56% | 47.37% | 31.96% | 85.14% | 88.49% | 85.30% |
| Ever | 7.39% | 9.67% | 7.43% | 2.03% | 3.02% | 1.13% |
| Current | 61.05% | 42.96% | 60.13% | 12.84% | 8.49% | 12.85% |
| Unknown | 0.00% | 0.00% | 0.48% | 0.00% | 0.00% | 0.72% |
| Number of screening |  |  |  |  |  |  |
| 1 | 62.12% | 25.93% | 56.49% | 73.99% | 17.14% | 56.73% |
| 2 | 23.38% | 26.87% | 25.51% | 17.57% | 28.13% | 27.03% |
| 3 | 11.19% | 24.39% | 14.69% | 7.77% | 21.25% | 14.18% |
| 4 | 3.31% | 22.81% | 3.31% | 0.68% | 33.49% | 2.06% |
| TB diagnosis |  |  |  |  |  |  |
| No | 99.55% | 99.81% | 99.52% | 99.66% | 99.95% | 99.90% |
| Yes | 0.45% | 0.19% | 0.48% | 0.34% | 0.05% | 0.10% |
| Charlson Comorbidity Index |  |  |  |  |  |  |
| 0 | 59.50% | 38.19% | 42.94% | 42.23% | 39.64% | 24.97% |
| 1 | 15.54% | 23.87% | 17.74% | 16.89% | 27.24% | 18.60% |
| 2 | 10.46% | 15.85% | 13.69% | 17.23% | 16.09% | 19.12% |
| 3+ | 14.50% | 22.09% | 25.63% | 23.65% | 17.03% | 37.31% |
