# Supplementary material for: Homelessness and mortality: gender, age, and housing status inequity in Korea
Source: Epidemiol Health. 2024 Sep 12;46:e2024076. doi: 10.4178/epih.e2024076 (PMC11826014; doi:10.4178/epih.e2024076)
Supplement: Supplementary Material 2. — Share of cause of death by gender and housing status [file epih-46-e2024076-Supplementary-2.docx]

## Supplementary Material 2. Share of cause of death by gender and housing status

|  | Total | | | Men (n=404) | | | Women (n=46) | | | |
| --- | --- | --- | --- | --- | --- | --- | --- | --- | --- | --- |
|  | *Rough*  *sleepers (%)* | *Facilities (%)* | *Jjokbang (%)* | *Rough*  *sleepers (%)* | *Facilities (%)* | *Jjokbang (%)* | *Rough*  *sleepers (%)* | *Facilities (%)* | *Jjokbang (%)* |  |
| Infectious diseases (A00-B89) | 5.1 | 4.0 | 4.1 | 5.3 | 4.1 | 3.9 | 0.0 | 3.5 | 7.1 |  |
| Neoplasm (C00-C97) | 7.6 | 22.4 | 18.2 | 6.6 | 22.7 | 19.2 | 33.3 | 20.7 | 7.1 |  |
| Endocrine diseases (E00-E99) | 2.5 | 2.5 | 5.9 | 2.6 | 2.3 | 6.4 | 0.0 | 3.5 | 0.0 |  |
| Mental disorders (F00-F99) | 3.8 | 1.5 | 1.8 | 4.0 | 1.2 | 1.9 | 0.0 | 3.5 | 0.0 |  |
| Neurological diseases (G00-G99) | 1.3 | 4.0 | 1.8 | 1.3 | 3.5 | 1.3 | 0.0 | 6.9 | 7.1 |  |
| Circulatory diseases (I00-I99) | 26.6 | 15.9 | 19.4 | 27.6 | 15.7 | 18.6 | 0.0 | 17.2 | 28.6 |  |
| Respiratory diseases (J00-J99) | 6.3 | 18.4 | 8.8 | 6.6 | 19.2 | 8.3 | 0.0 | 13.8 | 14.3 |  |
| Gastrointestinal diseases (K00-K93) | 8.9 | 7.0 | 14.7 | 9.2 | 5.8 | 16.0 | 0.0 | 13.8 | 0.0 |  |
| External causes (V01-Y98) | 10.1 | 7.5 | 8.2 | 9.2 | 7.6 | 9.0 | 33.3 | 6.9 | 0.0 |  |
| Not classified (R00-R99) | 16.5 | 8.0 | 10.6 | 17.1 | 8.7 | 9.6 | 0.0 | 3.5 | 21.4 |  |
| Infectious diseases (A00-B89) | 2.5 | 3.0 | 1.8 | 2.6 | 3.5 | 1.3 | 0.0 | 0.0 | 7.1 |  |
| Others | 8.9 | 6.0 | 4.7 | 7.9 | 5.8 | 4.5 | 33.3 | 6.9 | 7.1 |  |
