# Supplementary material for: Homelessness and mortality: gender, age, and housing status inequity in Korea
Source: Epidemiol Health. 2024 Sep 12;46:e2024076. doi: 10.4178/epih.e2024076 (PMC11826014; doi:10.4178/epih.e2024076)
Supplement: Supplementary Material 3. — Specific causes of death by gender and housing status [file epih-46-e2024076-Supplementary-3.docx]

## Supplementary Material 3. Specific causes of death by gender and housing status

|  | | Total (%) | Men (n=404, %) | Women (n=46, %) |
| --- | --- | --- | --- | --- |
| Cardiovascular (I20-I51) | | 10.9 | 10.4 | 15.2 |
| Cerebrovascular (I60-I69) | | 6.0 | 6.2 | 4.3 |
| Cancer | Lung (C33-C34) | 6.4 | 7.2 | - |
|  | Colon (C18-C21) | 1.8 | 2.0 | - |
|  | Liver (C22) | 2.2 | 2.0 | 4.2 |
|  | Gastric (C16) | 1.8 | 2.0 | - |
|  | Pancreatic (C25) | 0.7 | 0.7 | - |
| Liver disease (K70-K86) | | 5.8 | 6.2 | 2.2 |
| Hypertension (I10-I13) | | 2.0 | 2.2 | - |
| Pneumonia (J12-J18) | | 8.2 | 7.9 | 10.9 |
| Diabetes (E10-E14) | | 2.4 | 2.5 | 2.2 |
| Tuberculosis (A15-A19) | | 1.1 | 1.2 | - |
| COPD ^a^ (J40-J47) | | 3.6 | 4.0 | - |
| Not classified (R99) | | 5.8 | 6.2 | 2.2 |
| Others | | 41.3 | 39.4 | 58.7 |

^a^ Chronic obstructive pulmonary disease
